# Establishing the Medical Actionability of Genomic Variants

**DOI:** 10.1146/annurev-genom-111021-032401

**Published:** 2022-04-01

**Authors:** Katrina A.B. Goddard, Kristy Lee, Adam H. Buchanan, Bradford C. Powell, Jessica Ezzell Hunter

**Affiliations:** 1 Department of Translational and Applied Genomics, Center for Health Research, Kaiser Permanente Northwest, Portland, Oregon, USA; 2 Department of Genetics, University of North Carolina, Chapel Hill, North Carolina, USA; 3 Genomic Medicine Institute, Geisinger Health System, Danville, Pennsylvania, USA; 4 Genomics, Ethics, and Translational Research Program, RTI International, Research Triangle Park, North Carolina, USA

**Keywords:** actionability, medical actionability, clinical utility, evidence, intervention, genomic medicine

## Abstract

Actionability is an important concept in medicine that does not have a well-accepted standard definition, nor is there a general consensus on how to establish it. Medical actionability is often conflated with clinical utility, a related but distinct concept. This lack of clarity contributes to practice variation and inconsistent coverage decisions in genomic medicine, leading to the potential for systematic bias in the use of evidence-based interventions. We clarify how medical actionability and clinical utility are distinct and then discuss the spectrum of actionability, including benefits for the person, the family, and society. We also describe applications across the life course, including prediction, diagnosis, and treatment. Current challenges in assessing the medical actionability of identified genomic variants include gaps in the evidence, limited contexts with practice guidelines, and subjective aspects of medical actionability. A standardized and authoritative assessment of medical actionability is critical to implementing genomic medicine in a fashion that improves population health outcomes and reduces health disparities.

## INTRODUCTION

Clinical utility and actionability are related and important concepts. However, neither one has a well-accepted standard definition, nor is there a general consensus on how to establish clinical utility or actionability to the satisfaction of multiple stakeholders, particularly in the context of genomic medicine. Both concepts are centered around the general notion of determining whether an action or intervention will result in a benefit. Clinical utility is most broadly defined as “the usefulness of an intervention for, or in, clinical practice” (72, p. 377). However, definitions for clinical utility differ in terms of who takes the action (e.g., the clinician, the patient, or the patient’s family), who receives the benefit (e.g., the patient, the patient’s family, or society), what kinds of benefits are considered (e.g., a therapeutic effect, ending a diagnostic odyssey, or informing reproductive decision-making), how (or whether) the balance of benefits and risks are taken into consideration, whether an alternative is explicitly considered (e.g., comparative utility), and whether aspects of program implementation are included (e.g., availability, equitable access, or financial costs).

In our conceptualization of these terms that we present here, actionability is broader than clinical utility, incorporating a spectrum from clinical to nonclinical actions and taking into consideration different people who take the action, different recipients, and different benefits (32, 42). We make a key distinction between these terms by considering actionability at a person level (i.e., what is the usefulness of the intervention from a person’s perspective?) and clinical utility at a system level (i.e., what is the usefulness of the intervention from a clinical system’s perspective?). We further define medical actionability as the subset of actionability where the actions are taken in the clinical context. Medical actionability includes the known actions that a clinician could take to intervene that are specific to the condition under consideration, the evidence that these actions will avert a poor patient outcome due to a previously unsuspected high risk of disease, and consideration of risks or harms from taking these actions.

Genome sequencing and other molecular tests are informational and only indirectly impact health outcomes by influencing decision-making around the implementation of future actions or interventions (45).Thus, evaluation of the actionability or clinical utility of a test must consider the degree to which the test result first influences decision-making, and then must also assess whether further actions or interventions have a direct and beneficial impact on health outcomes. The overall decision-making and implementation of actions may be multistep, so quantifying actionability may require use of evidence from multiple sequential interventions. For example, subsequent steps may include a surveillance program (e.g., imaging or biochemical testing) that guides the initiation of downstream interventions (e.g., surgery or pharmacotherapy). A comprehensive evaluation of the evidence of all steps might include separate studies to evaluate the analytical validity of the imaging technique and to evaluate the effectiveness of surgery to prevent poor health outcomes.

The actionability or clinical utility of knowing genomic information produced by a test might vary depending on the timing. The test could come too late—the condition may have already progressed to a point where intervention is no longer effective or as effective as it could have been with earlier intervention. This is part of the rationale for screening newborns—to be able to intervene as early as possible in the disease course for conditions that typically arise soon after birth (77). As another example, carrier screening becomes less relevant to an individual once family planning and childbearing are completed (24). The test could also come too early—potential harms may be avoided by postponing learning about the information from the test, while potential benefits may still be realized, since the actions or interventions may not be initiated until years or decades into the future, if ever. This is part of the rationale for avoiding the prediction of risk for adult-onset conditions among children (64). As another example, the risk or burden of the intervention may change over time. For instance, the impact of bilateral salpingo-oophorectomy may be perceived differently after childbearing is complete.

The actionability of the information from a genomic test may relate to the timing of the onset of symptoms. If there are no preventive actions, testing while an individual is healthy or asymptomatic will not yield any clinical benefit. Even if there are preventive actions, the balance of risks and benefits of treatment for healthy individuals might be uncertain, particularly in the setting of opportunistic screening or population screening (11, 12, 21, 78). For instance, it may be unclear what to recommend for an individual at risk for a hereditary colorectal cancer syndrome depending on the overall health, age, and comorbid conditions for the individual. Similar considerations apply for reporting secondary findings, meaning findings that are not associated with the indication for diagnostic testing. The information from a test might impact clinical decision-making once symptoms have occurred, such as by informing prognosis or treatment choices. For instance, information from a pharmacogenomic test is generally relevant only if the individual needs one of the indicated drug therapies.

Greater clarity about the definitions of clinical utility, medical actionability, and actionability and clear thresholds for the evidence needed to establish them are important for the practice of medicine and decisions about use of genomic tests. Genomic medicine suffers from inconsistency in coverage decisions, disparities in access to tests, and a lack of clarity about what decision-makers want in terms of evidence. Peabody et al. (58) reported that lack of clinical utility data is the most commonly cited reason for the failure to receive favorable coverage and reimbursement decisions for molecular diagnostics. Inconsistency in guidelines and coverage decisions leads to practice variation, which can result in systematic bias and health disparities. Realizing the benefits from genomic medicine and achieving equitable health outcomes will not be possible without greater agreement about how to establish clinical utility and, more broadly, how to establish medical actionability.

In this review, we discuss the historical context of frameworks that have shaped our thinking about how to define actionability and medical actionability and the evidence needed to establish it. We present practical applications to illustrate the general significance of understanding medical actionability to society. We discuss the current and future challenges in establishing medical actionability and some proposed approaches to address those challenges.

## HISTORICAL CONTEXT

The practice of medical genetics, like other fields of medicine, is supported by clinical practice guidelines that are intended to promote high-quality and consistent care for patients. These guidelines are the best available source to identify potential interventions, or actions, that might drive the medical actionability of genomic information. Guidelines are produced by sources such as professional societies, national health systems, or a group of individuals designated (sometimes self-designated) as experts in the field.

However, guidelines can vary in many factors that impact quality, including reliance on expert opinion instead of evidence, identification of conflicts of interest, relevance to the population, and inclusion of updated sources. This variation can lead to inconsistency in recommendations, confusion, and bias. In 2011, the Institute of Medicine (now the National Academy of Medicine) proposed eight standards to promote trustworthy practice guidelines: transparency, management of conflict of interest, systematic review of the evidence, rating the strength of guideline recommendations, clearly articulating recommendations, external review, and updating (33). It is an ongoing effort for organizations to address these standards in all practice guidelines.

The evaluation of genomic applications for clinical practice has leveraged a long history of evaluation frameworks for other kinds of medicine, and clinical utility is a key component of these frameworks. A framework proposed for diagnostic imaging is frequently used as a model since, similar to genomic applications, the imaging itself has only an indirect impact on health outcomes (30). Wilson & Jungner (87) proposed a general set of principles for population screening that has been adapted for genomic applications in both newborn screening (2) and adult screening (47) populations. The US Task Force on Genetic Testing (81) used clinical utility in a narrow sense and emphasized interventions that would prevent disease or improve health outcomes for individuals. In this narrow framing, a diagnosis alone would be insufficient, and additional evidence would be needed to demonstrate disease prevention or changes in treatment, prognosis, or disease management.

The ACCE framework (13, 37)—which stands for the four components of analytic validity, clinical validity, clinical utility, and ethical, legal, and social issues—was developed in the context of screening and diagnostic testing using genomic applications, and it builds upon prior work by Wald & Cuckle (83) for screening and diagnostic testing more generally. The Evaluation of Genomic Applications in Practice and Prevention (EGAPP) initiative explicitly adopted this framework as part of its efforts to implement a rigorous, evidence-based process to evaluate genomic applications (76), in addition to using methods from the US Preventive Services Task Force (80). Lessons learned from this early experience highlighted some of the important challenges facing the field today (27). Namely, this is a rapidly evolving and dynamic field of study with manifold conditions, most of which are individually rare. Thus, evidence is sparse, and the studies are of lower quality and smaller than those typically found in other areas of medicine. Relevant stakeholders have different professional interests and norms, and there are fundamental differences in values and outcomes that are considered important.

## PRACTICAL APPLICATIONS AND GENERAL SIGNIFICANCE OF ESTABLISHING ACTIONABILITY TO SOCIETY

### Broad Versus Narrow Definition of Actionability

**Figure 1:**
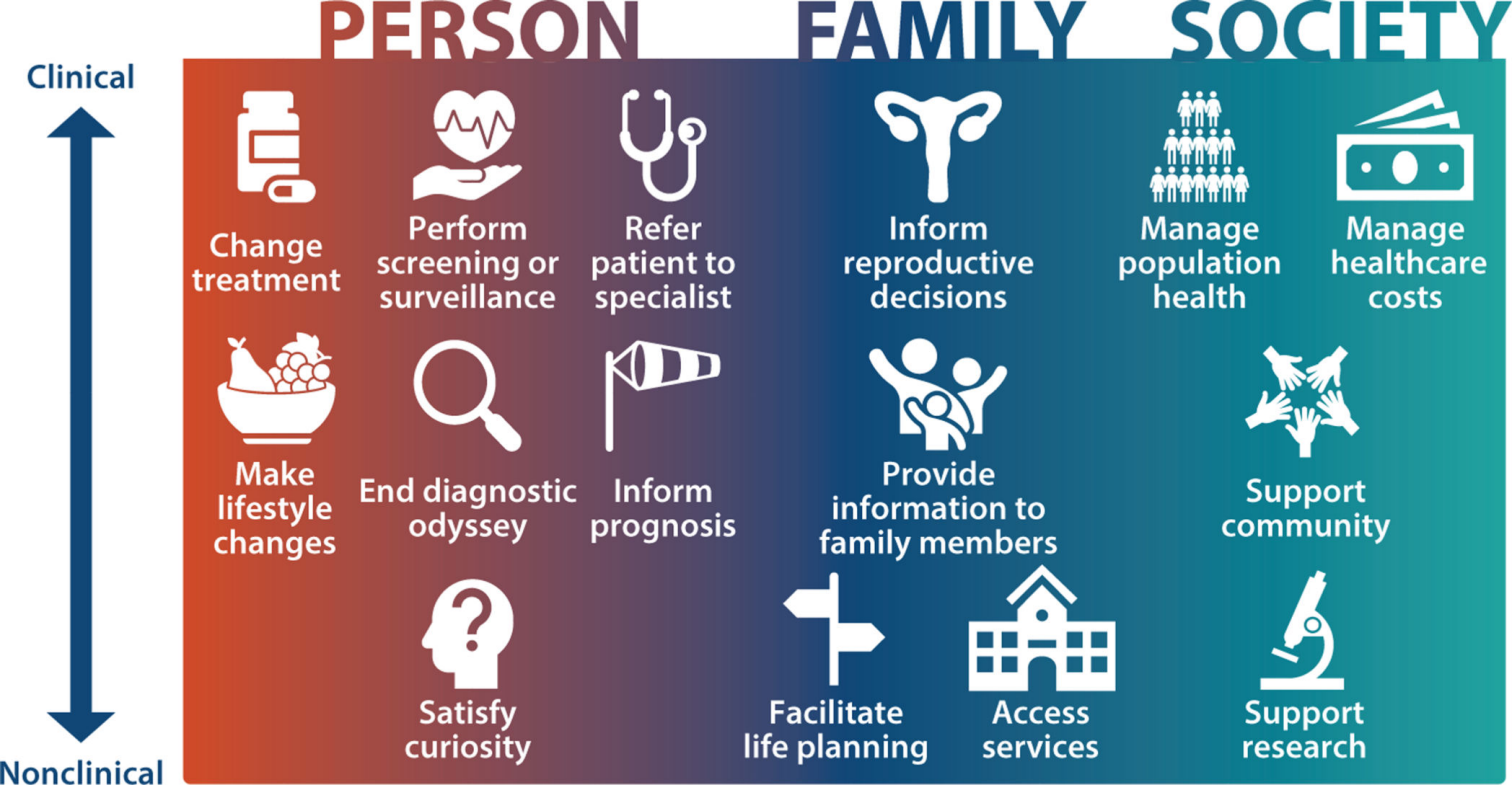
The spectrum of actionability, which includes benefits for the person, the family, and society.

Most stakeholders recognize that a broad range of potential actions can result from acquiring genomic information (43, 69) (Figure 1). A holistic view of a person includes recognition that life choices and family context are important aspects of that person’s health. Individuals may perceive benefits that are indirectly related to physical or mental health. In defining medical actionability, the delivery of genomic services through the healthcare system has a strong influence on valuing clinical actions more heavily in decisions about coverage and reimbursement for genomic testing. Actions that must be taken by a clinician are the easiest to justify in the context of healthcare spending. Benefits that accrue to the individual patient are also most readily accepted as medically actionable, particularly for healthcare systems funded mainly through private insurance, such as those in the United States, compared with public healthcare systems that cover a population. However, even for systems that focus on the health of the population as a whole, there are complications to managing benefits for families or society that may preclude implementation of testing to achieve these benefits. We review the full spectrum of actionability, recognizing that the threshold for actionability differs across the perspectives of individuals, families, clinicians, payers, and other stakeholders.

Changes in clinical management to improve the morbidity and mortality associated with a condition can take a variety of approaches. There may be preventive actions, such as risk-reducing medications for individuals with hereditary cardiomyopathies or arrhythmias, or avoidance of certain kinds of anesthesia for individuals with malignant hyperthermia. Even if the condition cannot be avoided, early diagnosis or detection could lead to an improved health outcome. For instance, monitoring aortic dilation for individuals with Marfan syndrome may enable timely intervention to avoid aortic rupture. Once a condition has been diagnosed, clinical management could still change based on genomic information if there are different treatment options available. For instance, in the treatment of hypercholesterolemia, the therapeutic target is lower for individuals with familial hypercholesterolemia compared with other forms. In the treatment of diabetes, individuals with maturity-onset diabetes of the young are often misdiagnosed with another form of diabetes, and thus are not receiving the most effective therapy for their condition.

Health outcomes may also be improved by referral to a specialist or a multidisciplinary specialist team, even if the management options do not differ. Specialists with a broader and deeper knowledge about a condition may be better able to coordinate care. For example, patients with neurofibromatosis type 2 have a significantly lower risk of mortality when their care is managed at a specialty center with a multidisciplinary team compared with those who are treated at non-specialty centers (28).

Actionability can also be on the border between personal and medical. A clinician may recommend lifestyle changes related to diet, exercise, smoking, or sun exposure, but these changes are generally implemented outside of a healthcare environment. Ending a diagnostic odyssey can have great personal benefit for individuals and their families even when there are no treatment options available (52). It can provide closure to their search for an answer, information about the natural history of the condition, and access to services, as well as linking families to patient support groups for others with the same condition or providing them with opportunities to participate in targeted clinical research. From the health system perspective, there may still be benefits even if the genomic information does not guide patient management to improve health. Individuals with complex, undiagnosed conditions often undergo a long series of healthcare visits and procedures. The genomic test may be a less expensive alternative to these medical encounters, extensive laboratory testing, and procedures, and avoiding these additional measures may also prevent potential harms. An informed prognosis may have a great psychological benefit for individuals and their families and help the clinician to tailor management, even if it does not change the health outcome.

Some aspects of actionability relate primarily to the personal choices or options of individuals and their families and may happen outside of the healthcare system. For instance, knowing about hereditary risks for adult-onset conditions, such as Alzheimer disease or frontotemporal dementia, may allow individuals and families to make different life choices or to plan differently for their future. Preconception and prenatal carrier screening offer potential parents the opportunity to consider different options as part of their reproductive planning, including the choice to adopt, to use donor gametes or embryos, or to not have children, as well as the choice to use different reproductive technologies, including preimplantation genetic diagnosis or prenatal diagnosis. Access to some educational or social services may be supported by a specific medical diagnosis, so genomic information may expand the available options to the individual and family, such as accommodations or services for autism, visual impairment, or hearing loss.

For some aspects of actionability, the benefits for the individual may be unclear or may depend on the circumstances. Informing family members of a hereditary risk may or may not have a direct benefit for the individual. For instance, in the context of secondary findings from germline testing in children, informing families about risks for adult-onset conditions, such as hereditary breast and ovarian cancer, may allow a parent the opportunity to better manage their risk and thus directly benefit the child by promoting the survival of the parent (86). In other contexts, informing family members has the primary benefit of allowing them to manage their own risk and life decisions. Likewise, genomic testing primarily to satisfy curiosity could also have some potential benefits for the individual (66, 90). It could allow the individual to feel more in control, to mentally prepare for the future, and to cope with future health risks.

Finally, sometimes actionable genomic information has primarily an altruistic or societal benefit. Obtaining a clear genomic diagnosis may enable individuals to enroll in research studies, which has the potential to inform treatment for future individuals with the condition. Individuals may report personal utility from this altruistic contribution to society. In addition, particularly for very rare conditions, a clear genomic diagnosis can help the community understand the phenotypic spectrum and natural history associated with the condition. Through registries such as Genome-Connect (67),patients, families, and advocacy groups can join together to improve knowledge and interpretation of genomic information by sharing their deidentified genetic information and receive updates as information evolves over time. Although, at present, this information might not have a clear clinical benefit for the individual, these altruistic actions may lead to a clear clinical benefit in the future.

### Actionability Across the Life Stages

**Figure 2:**
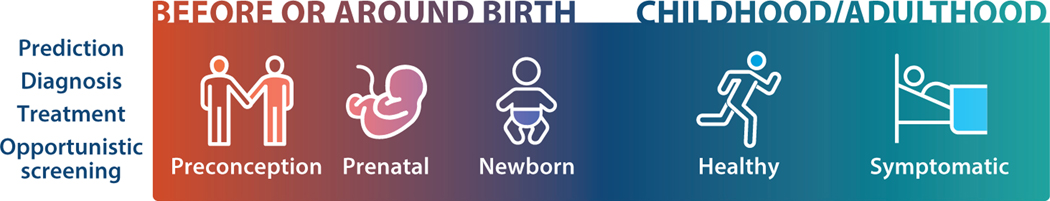
The spectrum of actionability across the life course. Within each stage of life, genomic tests can be actionable for different applications, including prediction, diagnosis, and treatment.

The concept of actionability has developed separately across life stages and applications of genomic tests (Figure 2). The unique circumstances of the clinical context may differ, whether it is to predict a future health risk, to diagnose a condition, or to guide a treatment plan. In addition, the generation of genomic information allows for opportunistic screening for secondary findings unrelated to the initial reason for testing. The relative importance of factors in specific contexts drives variation in the specific criteria and considerations that make a genomic test actionable in each setting.

#### Adult.

For adults, we discuss the actionability of genomic tests separately in the context of risk prediction, diagnosis, and treatment, since the meaning of or opportunity for actionability may be different depending on the context.

##### Risk prediction and opportunistic screening.

For adults, risk prediction is relevant primarily for undiagnosed individuals who are either asymptomatic and not yet showing signs of a condition or symptomatic but do not yet have a definitive diagnosis of a hereditary condition. With the advent of lower-cost DNA sequencing technologies and more widespread use and availability of genome sequencing for indication-based diagnostic purposes, opportunistic screening to identify actionable secondary findings has become an important screening strategy.

As part of our work for the Clinical Genome Resource (ClinGen) (61) Actionability Working Group, we have defined and implemented a process to evaluate medical actionability for this clinical scenario (42). Our work builds upon prior efforts to define a rapid and systematic evidence review process and to define a semiquantitative metric to rate the level of actionability through an expert review process (7,32,82).We consider and assess four components of medical actionability: the severity of the condition, the likelihood of the health outcome, the effectiveness of the intervention, and the nature of the intervention, the last of which captures potential adverse effects of the intervention as well as its burden and acceptability. Each of the four components is rated on a four-point scale, and the quality of evidence for the likelihood of the health outcome and the effectiveness of the intervention is evaluated. The severity of the condition and the nature of the intervention are inherently subjective and thus are not evaluated for quality of evidence. The total score is a simple linear combination of the individual component scores and can range from 0 to 12. These assessments are available through the ClinGen website (https://clinicalgenome.org). The association between the gene(s) and the condition(s) or the pathogenicity of variants within the gene(s) is evaluated using standardized protocols as part of ClinGen through gene curation and variant curation efforts.

In terms of the severity of the condition, we consider a condition to be most medically actionable for risk prediction when the initial manifestation can be sudden death, which may occur, for instance, for some arrhythmias that do not otherwise present clinically. Morbidity and mortality are then considered on a scale of low, moderate, and high. Phenotypic traits that are not related to health outcomes are not actionable on this scale (e.g., the ability to roll one’s tongue or taste cilantro).When evidence on the effectiveness of the intervention to impact morbidity and mortality is not available, we may consider evidence for other health outcomes, such as improvement in intermediate clinical biomarkers or signs (e.g., blood pressure or cholesterol level), or functional outcomes, such as impact on daily living activities. We use a narrow, clinically focused definition for the interventions or actions that are considered, including changing treatment options, performing screening or surveillance, and referring the patient to one or more specialists, and circumstances to avoid, such as the use of certain types of anesthesia for patients with malignant hyperthermia or radiation therapy for patients with Li–Fraumeni syndrome.

The American College of Medical Genetics and Genomics (ACMG) has established guidelines for proactively seeking and reporting medically actionable secondary findings; these are currently in their third iteration (34, 46, 54). The ACMG uses a relatively narrow definition for medical actionability that includes actions at the clinical end of the actionability spectrum, and in many cases considers the results of evidence curation performed by the ClinGen Actionability Working Group. In a research context, versions 1.0–3.0 of the ACMG Secondary Findings list have been used worldwide, and large studies have found a frequency of approximately 2% for medically actionable secondary findings on this list (26,38,82,92).However, professional organizations from other countries provide conflicting guidance and do not recommend opportunistic screening and disclosure of medically actionable secondary findings, citing the need for a more cautious approach to evaluate the balance between risks and benefits in low-risk populations, consideration of costs in relation to other healthcare expenditures, and development of further evidence (11, 21, 78).

Despite the very narrow definition of medically actionable findings that is incorporated into practice guidelines, different stakeholders have a much broader view of what is relevant. Delanne et al. (23) conducted a literature review on the perspectives of participants (or parents), healthcare providers, and general members of society on the types of secondary findings that they would want to receive once patients have provided informed consent. They distinguished between actionable (meaning that therapeutic or preventive options are available, which is akin to how we define medical actionability) and nonactionable secondary findings. Many findings that are considered nonactionable according to this definition would fall within the spectrum of actionability as we have defined it, but outside of medical actionability (Figure 1). Although for all groups a higher percentage wanted (medically) actionable secondary findings provided compared with (medically) nonactionable secondary findings (92% versus 70% of respondents), a substantial majority of all groups wanted results for (medically) nonactionable secondary findings.

On the horizon, population screening to identify individuals at risk for medically actionable conditions is under investigation in research settings. At present, this preventive screening approach is not recommended by any professional organizations, including the ACMG and the US Preventive Services Task Force. Because the scale of a population-based public health approach requires a less resource-consuming consent and individualized decision-making process, further work is needed to contextualize the balance of risks and benefits at the population level (12). Importantly,this work will need to include ensuring equitable access to genomic information to avoid exacerbation of existing health inequities.

##### Diagnosis.

Diagnostic testing is relevant for individuals who have already developed signs and symptoms of a condition. While monogenic disorders are individually rare, they are collectively common and impact approximately 1.5–6.2% of the global population (29). The morbidity and mortality burden from these conditions is disproportionate to their prevalence. However, traditional medical genetics diagnostic evaluations reach a diagnosis in less than half of cases. The time to diagnosis from the initial recognition of signs or symptoms (i.e., the diagnostic odyssey) can be as long as 5–30 years under current standards of care (91), and the diagnostic odyssey can include multiple specialist visits, invasive procedures, laboratory and genetic tests, and imaging studies.

Patients can experience barriers to receiving a diagnosis for numerous reasons. Given that there are approximately 6,000–8,000 rare genetic conditions, some of which are exceedingly rare, providers may lack familiarity and may not be expected to encounter a single patient with a particular diagnosis in their entire career. Rare diseases can mimic more common conditions or have substantial genetic heterogeneity or atypical presentations (68, 79).

Underrepresented minorities may experience additional barriers to receiving a diagnosis (29). A genetic condition might not be suspected because of limited definition and cataloging of dysmorphology in underrepresented populations, and developmental concerns may be attributed to a nongenetic cause such as adverse childhood events. Underrepresented minorities are also less likely to receive a referral to specialty services, and providers may instead prioritize addressing social determinants of health. Even if a referral is made and accessible, families may be unable to complete the referral because of the complexity of the clinical service.

A molecular genetic diagnosis can bring numerous potential benefits even if it does not inform the management plan. For conditions with poor prognosis, a diagnosis enables providers to appropriately counsel families on the goals of care. There may be economic and psychological benefits, such as relief of distress from knowing the diagnosis, and the diagnosis may support access to social support groups and services. Obtaining a diagnosis may validate health concerns of the family that may have been initially dismissed by healthcare providers (16,73).In a recent study of perspectives of US private payers, most (9 out of 14) viewed ending a diagnostic odyssey as a valid reason for clinical utility, but still considered factors relevant to the healthcare system to be part of this perspective, including stopping the need for other medical evaluations and informing condition-specific medical management (65).

##### Treatment.

After a person has been diagnosed with a specific condition, genomic information may be relevant to guide treatment decisions. For example, gene therapy that has been approved by the US Food and Drug Administration (FDA) is available for patients with confirmed biallelic *RPE65* retinopathy. For patients with hereditary transthyretin (*TTR*)–related amyloidosis, the FDA has approved pharmacotherapy with TTR-stabilizing agents only for TTR cardiac amyloidosis (70). The Clinical Pharmacogenomics Implementation Consortium has developed a methodology and produced clinical practice guidelines for gene–drug pairs that include recommended prescribing actions (18,62).In this case, actionability is narrowly defined by recommended actions, which may include choosing among alternative therapies or among different dosing strategies.

Although the majority of this review has focused on germline applications, both germline and somatic alterations are relevant to health outcomes in the context of cancer. Tumor genome sequencing to identify somatic genomic changes can also inform management for patients, but similar considerations must be taken into account in the assessment of clinical utility and actionability. One group has defined a concept of clinical significance, which is related to actionability, as part of an evaluation framework for genomic variants related to cancer (57). This concept includes the type of treatment response, the ability to type or subtype cancer, prognostic information on outcome, and information on the type of biological or functional change. These assertions are stored as part of the Clinical Interpretation of Variants in Cancer (CIViC) knowledge base (20).

#### Pediatric.

In the pediatric context, many of the same considerations regarding risk prediction, diagnosis, treatment, and opportunistic findings apply as described above for adults, but numerous authors have also raised additional considerations. There is no consensus on when or whether to disclose findings in recommendations across American, Canadian, and European practice guidelines in the pediatric context (11, 40, 54).

##### Risk prediction.

Although most stakeholders agree on medical actionability at the clinical end of the spectrum, where there are surveillance, preventive, or treatment options available that can significantly improve health, the timing of intervention initiation is an important consideration in addition to the timing of the onset of symptoms. Disorders that typically manifest symptoms during childhood are more likely to be considered medically actionable in the pediatric context. If it is beneficial to initiate the intervention in childhood, then even typically adult-onset conditions might still be actionable in children. For instance, in classic familial adenomatous polyposis, the average age of patients presenting with symptoms is 35.8 years. However, colorectal adenomatous polyps begin to appear, on average, by age 16. Thus, while cancer occurs only rarely (estimates range from 0.2% to 1.3%) in patients with familial adenomatous polyposis under age 20, surveillance is recommended starting at age 12–14 to guide the timing and type of prophylactic surgery (55).

Particularly in the context of risk prediction for adult-onset conditions, defining what is in the best interests of the child can be complex and controversial, and decisions for and by children can have implications for the remainder of their lives (9). Parental interests may not align with the child’s interests, and disclosure of adult-onset conditions to children could lead to potential harms, including overprotective parenting, preferential treatment within the family, and violation of the right to an open future (10). There is also often more limited or poor-quality evidence about the effectiveness of interventions in children to prevent adult-onset conditions (63). Risk information about adult-onset conditions may be of more immediate benefit to a parent (8, 9, 19, 51) if the inherited risk factor is also present in one of the parents. However, some have argued that disclosing the risk information may also benefit the child by preserving the family unit (10, 63). In addition, if adult genomic screening programs are not available, opportunistic disclosure of adult-onset risk information to children may be the only chance for them to learn about their risks and act on this information later in life.

##### Diagnosis.

Ending a diagnostic odyssey can bring closure to a family, even if the diagnosis is for a condition with a poor prognosis. Genetic diagnosis can account for seemingly unrelated symptomsorpredictsymptomsthathavenotyetoccurredbutmightbeexpectedbasedonatypical progression of the condition. Conditions that are vague and nonspecific (e.g., developmental delay or seizure) can progress in different ways, and a definitive diagnosis will provide a clearer picture for the future course of disease. Stivers & Timmermans (75) focused on the benefits of ending a diagnostic odyssey for parents of a child with a disability. In particular, they discussed how genomic information can help address parents’ feelings of guilt and blame for causing the child’s disabilities, stating that “geneticists...consistently offered a firm, unambiguous and conclusive exoneration of the parents” (p. 1548), especially when the genomic variant is de novo.

Genome-scale sequencing can achieve a molecular diagnosis for 25–40% of patients. One study identified molecular diagnoses for 29% of patients who were late in their diagnostic odyssey and had already had prior clinical and molecular investigations (68). The most common reasons that the condition was not diagnosed sooner were significant genetic heterogeneity (defined as more than three genes for a particular disorder) and atypical presentation. The costs of a diagnostic odyssey are highest in the first year and then remain stable for numerous subsequent years, indicating that families of undiagnosed individuals continue to pursue a diagnosis over long periods of time (25). One cost-effectiveness study in infants overwhelmingly supported the use of exome sequencing as a first-line test early in the diagnostic odyssey; compared with standard evaluations, this approach had more than triple the diagnostic rate for one-third the cost per diagnosis (74).

##### Treatment.

We do not note new issues related to treatment in the pediatric setting aside from those described above for the adult context.

#### Before or around birth.

The timing of testing very early in life, before or around birth, requires special consideration for actionability because of the unique risks and benefits during this period. In this life stage, similar to the adult and pediatric contexts, we consider genomic testing for risk prediction, diagnosis, and treatment.

##### Preconception or prenatal carrier screening.

Carrier screening is a form of risk prediction to assess the risk of autosomal recessive or X-linked conditions in the next generation rather than for the individual(s) or couple(s) undergoing testing. Approximately 2–4% of couples are estimated to be at risk of having an affected pregnancy (15). Actionability in the context of carrier screening refers to reproductive decision-making, with more options available to at-risk couples when screening is performed in the preconception period rather than in the prenatal period. Until recently, only a handful of conditions were routinely recommended and evaluated for carrier screening, and screening programs may be offered to a limited group of high-risk populations based on ethnicity or family history. The availability of genome and exome sequencing has broadened interest in and opportunities for expanding carrier screening programs to be pan-ethnic (or universal), to be more comprehensive in the variants that are evaluated within genes, and to include more genes and conditions as part of the screening.

Although most professional statements and recommendations broadly follow similar considerations, there are differences in the thresholds of what is considered actionable for carrier screening (1,35,41,88). The broad considerations are the carrier frequency, the severity of the condition, and limitation on life span. There is tremendous variation in the implementation of carrier screening programs in countries across the world (24), reflecting local factors such as differences in carrier frequency, the organization of healthcare, and cultural and religious factors. Some recommend a pan-ethnic approach to increase equity, reduce the chance of stigmatization, and increase identification of at-risk couples (35,41,59). For instance, in a large study of more than 381,000 individuals in the United States, 81.6% of carriers of conditions associated with Ashkenazi Jewish ancestry did not report this ancestry (85). In this same study, 44.0% of carriers for cystic fibrosis had a genomic variant that was not on the standard genotype panel. For pan-ethnic screening programs, carrier frequencies are expected to be relatively high, such as 1 in 100 (1) or 1 in 200 (35), because the chance of missing an at-risk couple becomes extremely small as the carrier frequency declines (6, 36). There is no agreed-upon definition for the severity of the condition. To address this concern, one group published an algorithm to define four categories of severity using factors such as shortened life span, intellectual disability, impaired mobility, and sensory impairment, with severity being modified by factors such as the availability of treatment and variable expressivity of the condition (50). Genetic counselors and medical geneticists then applied this algorithm to classify the severity of 176 conditions (3). However, other stakeholders, such as at-risk parents or payers, may have a different viewpoint about thresholds to use when considering the severity of a condition (3, 24). Some prospective parents may value a lower threshold and want to be informed of the risk for an affected offspring in order to prepare for the birth of their child, even if that information would not alter their reproductive decisions (48).

Multiple large studies that have assessed carrier screening programs indicate that the majority of at-risk couples do take action (14, 31, 44). In addition, a modeling study using data from more than 60,000 patients and more than 200 at-risk couples found that preconception carrier screening using a 176-condition panel would reduce the affected birth rate and would be estimated to be cost-effective (i.e., less than $50,000 incremental cost per life year) compared with either no screening or limited screening programs that include only cystic fibrosis and spinal muscular atrophy (5).

##### Newborn screening.

Newborn screening allows for timely diagnosis and treatment of conditions before significant, irreversible damage occurs. There is substantial variation in newborn screening programs worldwide (77).As an example of the kinds of considerations and evaluation criteria used to determine which disorders to include in newborn screening, we focus on the program that we are most familiar with: the Recommended Universal Screening Panel (39) in the United States. In this setting, newborn screening is implemented as a public health–based population genetic screening program using evaluation criteria that were established by the ACMG (84). There are numerous aspects of the evaluation criteria that go beyond our topic of medical actionability, including factors such as program implementation, cost, and availability of treatment. Thus, we focus on only a portion of the criteria to illustrate shared concepts.

Clinical characteristics of the condition are considered, including the incidence, the burden if left untreated, and the timing of early initiation of signs and symptoms. The intervention is evaluated for its potential to prevent negative consequences, including mortality, through early diagnosis and treatment, and for the simplicity of the intervention. Benefits of early intervention are considered from the perspective of the individual, the family, and society. While research studies have explored the technological advances in genome or exome sequencing, so far sequencing has demonstrated insufficient sensitivity and specificity to serve as a primary screening method in place of conventional approaches (89).As more gene-targeted therapies are developed (e.g., as for spinal muscular atrophy),the promise of newborn screening (however performed) is expected to expand.

Milko et al. (53) proposed a framework to evaluate medical actionability in the context of newborn screening for genome-scale sequencing. This framework consists of many of the medical actionability domains discussed above, including the severity of the condition, the likelihood of the health outcome, the efficacy of the intervention, the acceptability of the intervention, and a rating on the availability of evidence. DeCristo et al. (22) applied a similar framework of medical actionability to 309 conditions on one or more of fourcommercial panels for neonates.Theyfound substantial heterogeneity in the content of the panels, with only 74 genes (23.9%) included on all four panels. In addition, 82 genes (26.5%) listed on one or more panels were deemed inappropriate for newborn screening using the proposed metric.These findings indicate that frameworks for medical actionability could be leveraged to improve the consistency of medical actionability–based decision-making related to the return of findings in the newborn context.

## FUTURE OPPORTUNITIES AND CHALLENGES

Looking ahead, there are several areas where further development would support the ability to assess the medical actionability of genomic information. These include addressing gaps in the evidence and in the data model (e.g., developing a structured terminology that is machine interpretable), widening the contexts for which practice guidelines are available, improving uptake of best practices in developing practice guidelines, incorporating multiple stakeholder perspectives for subjective aspects of actionability, and exploring alternatives or modifications to the model for establishing actionability.

### Gaps in Evidence

Mendelian conditions are rare, so the existing evidence is typically based on small numbers of highly ascertained individuals who are identified because of clinical characteristics. Thus, decision-makers are left with the choice of making no decision for lack of evidence, making decisions based on evidence with a high degree of uncertainty, or making decisions based on evidence extrapolated from related conditions. Particularly when compared with areas of medicine where the standards for and availability of evidence are much higher, these circumstances for genomic medicine lead to inconsistency in decision-making.

There are some domains of medical actionability where current evidence is particularly limited. Data on the penetrance or likelihood of particular health outcomes are often missing or biased due to ascertainment based on phenotypic presentation. The risk of individuals ascertained through opportunistic screening or population screening programs is unclear. For the few conditions where penetrance has been estimated from unselected populations, the penetrance is typically lower compared with the estimates based on phenotypically ascertained families, presumably because there are additional environmental or polygenic factors that affect risk in heavily impacted families but are not present in all families. Thus, the available data for most conditions overestimate the level of risk. For genetically heterogeneous conditions, evidence may be uneven across genes, especially if one gene is more frequently the cause of the condition compared with the other genes.

**Figure 3:**
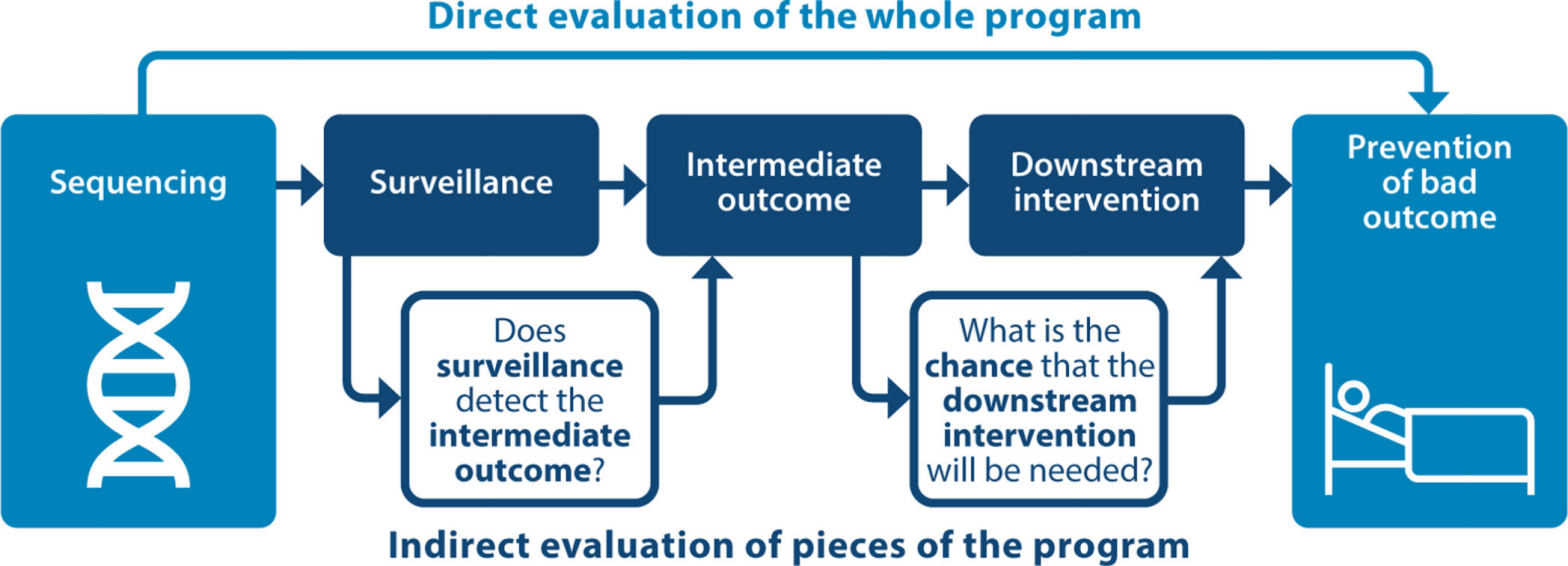
An analytical framework for answering the following question: Does sequencing genes for conditions that would then be monitored by a surveillance program reduce the risk of a bad health outcome compared with having no surveillance program? In some cases, there can be direct evidence about the program’s effectiveness (*top*); if there is no direct evidence, then individual pieces of the program may provide indirect evidence (*bottom*).

Data can also be limited regarding the effectiveness of interventions. In many cases, we do not anticipate that new evidence is forthcoming, or it would be considered unethical to not offer an intervention even though it is not evidence based. For example, evidence regarding the effectiveness of dietary management to limit protein intake in individuals with citrullinemia is limited to case studies and descriptive studies. Better quantification of the effectiveness of dietary management to minimize morbidity and mortality is not forthcoming since clinical trials of dietary management versus no dietary management would be unethical. For conditions where the intervention is surveillance, we often do not have direct evidence of the impact of surveillance programs on health outcomes and must rely on stringing together an indirect chain of evidence to evaluate the potential impact of the program (Figure 3). Often, it is difficult to assess the effectiveness of avoiding certain situations or activities (e.g., contact sports in individuals with Marfan syndrome) because there are no randomized studies of this type of intervention.

Given that these gaps in evidence are likely to persist, we recommend a few approaches to allow the field to move forward while acknowledging these limitations of the evidence. First, we recommend using a systematic approach that provides clarity and transparency about the availability and quality of evidence. For instance, in the actionability semiquantitative metric we use in ClinGen, we grade the evidence and document the tier of evidence as part of our metric (42). Second, we recommend extrapolation from other populations when evidence is not available for individuals with a specific condition, along with transparency on when extrapolation is used. For instance, while there are numerous studies of the effectiveness of sunscreen to reduce risk for skin cancer in the general population, there is no such evidence on sunscreen effectiveness in individuals with pathogenic variants in genes associated with nevoid basal cell carcinoma syndrome (71). Finally, solutions are needed to generate data and inform decision-making, particularly for rare conditions. One example of an innovative approach is the Early Check study, a partnership between a research program and a state newborn screening program to investigate rare conditions with significant advances in treatment (4).

### Availability and Variability of Practice Guidelines

Our approach to assess actionability relies on the existence of practice guidelines rather than assessment of the primary literature. The vast majority of existing guidelines assume the patient is symptomatic, which may not be relevant for clinical scenarios involving undiagnosed individuals, such as in opportunistic or population screening. Even if practice guidelines do exist for the appropriate clinical context, there can be a range of recommendations that might conflict with each other across different guidelines. As noted above, guidelines vary in factors that impact quality, including their reliance on evidence versus expert opinion, transparency of methodology, and attention to bias. Especially when evidence is emerging on a new intervention, there may be upstream indicators, such as FDA approval of a novel therapeutic, that have not yet been incorporated into practice guidelines. We recommend continued adoption of the standards recommended by the National Academy of Medicine.

### Gaps in the Data Model

We use ontologies to share information using a common vocabulary, including the need for machine-interpretable terminology. For the field of genomics, numerous ontologies exist for genes, phenotypes, and clinical conditions. While ontologies are emerging for interventions (17, 49),the field would benefit from greater development in this area. An intervention ontology would support consistency in the naming of interventions across clinical domains and clarity in defining the interventions. This is particularly challenging for complex conditions with multiple interventions that may be clustered together under a general heading, such as multidisciplinary care. Often, the evidence is based on a program that provides a constellation of interventions (e.g., surveillance for multiple organ systems in cancer predisposition syndromes) rather than evaluation of evidence for each intervention individually. An intervention ontology would support searching in actionability databases to view different clinical domains that share a common intervention. It would also enable clustering of related interventions to compare or consider actionability in related scenarios. For instance, surveillance can be subdivided into categories of imaging, biomarkers, and physical examinations, which can each be further subdivided into more specific interventions within each category.

### Subjective Aspects of Actionability

Even within the context of a very narrowly defined medical actionability, several aspects of the assessment are necessarily subjective and not evidence based. In particular, the way that an individual interprets the severity of a condition depends at least somewhat on personal experience of the impact of condition-related morbidity and mortality. For instance, sensory impairment or metabolic disorders may be perceived as a manageable hurdle for some people and a debilitating obstacle for others. We do not have existing scales to measure the perception of the severity of a condition, and perception might differ among experts, laypeople, and affected individuals. Perception of severity may even change within a person—for instance, the anticipation of the condition before it develops may differ from the experience of the condition after manifestations develop.

Likewise, the nature of the intervention—meaning the perception of acceptability, tolerability, burden, and risk—is also quite subjective. We do not have existing objective measures to quantify these features of the intervention, and we also expect the perception of the nature of intervention to differ between experts and laypeople, and especially depending on whether the person has experienced the intervention (56). Furthermore, perceptions of the nature of the intervention may vary from person to person based on personal preferences (e.g., avoidance of contact sports) and behaviors (e.g., avoidance of smoking for a nonsmoker versus a current smoker) as well as across the life spans of individuals (e.g., the risk or burden of an oophorectomy before versus after menopause). This area would benefit from further development of standardized and validated measures and scales, as well as systems to capture this evidence from a variety of stakeholders.

### Models for Assessing Actionability

In ClinGen, we have developed one model to systematically assess medical actionability, but there are alternatives to this model that could be explored in other settings. For instance, although we assess four components—the severity and likelihood of the condition and the effectiveness and nature of the intervention—it is not clear how to combine these components into an overall assessment of medical actionability. Adding the components to obtain a total score assumes a linear combination where each component has equal weight, which can sometimes lead to counterintuitive results. One example is hereditary diffuse gastric cancer associated with *CDH1*, where there is a high likelihood of gastric cancer and a significant risk of morbidity, and where gastrectomy (i.e., removal of the stomach) is the sole effective intervention to reduce the risk for developing gastric cancer, which surveillance is not effective at detecting at early stages. However, because gastrectomy is quite impactful and burdensome, the total score is only 8 out of 12. Interventions for other conditions, such as surveillance for endometrial cancer in Lynch syndrome, receive a similar total score of 8 out of 12 despite being less effective. Therefore, we have developed a process for making an assertion of medical actionability based on a rubric that prioritizes effectiveness and is not a linear combination of subscores, although other approaches could be explored.

As another example, there could be different approaches to structuring the expert panel. The expertise on the ClinGen Actionability Working Group includes primarily medical geneticists and genetic counselors, although we do have ad hoc members with particular expertise, such as cardiology, who join the group for certain topics. A potential alternative is to have multiple panels (e.g., each panel focuses on a subset of genes or conditions within their area of expertise), which aligns with how other ClinGen working groups who assess gene–condition validity or variant pathogenicity are structured. Another option is to have a broader representation of expertise with different areas of medicine represented beyond medical genetics. Efforts to evaluate these configurations would need to address the logistical challenges of assembling, training, managing, and providing evidence synthesis for multiple groups. The configurations would also need to balance the potential for engaging broader perspectives with the risk of inconsistency in how the experts consider different aspects of the evidence across different topics.

## LIMITATIONS

Our approach to assess medical actionability leaves out many important considerations that must also be evaluated as part of a comprehensive determination regarding when and whether to offer genomic testing. We recognize that it is equally important to ensure that the gene–condition association is valid and that clear and consistent criteria are used to classify variants on the spectrum from pathogenic to benign. Within ClinGen, there are separate processes to assess gene–condition validity and variant pathogenicity, and thus we are able to isolate our assessment of medical actionability from these other critical components. In addition, once genes are determined to be actionable, many logistical challenges must be considered as part of a comprehensive assessment. The analytic validity of a specific test platform must be evaluated along with practical issues such as how to develop an efficient analytical pipeline (60).A full assessment will need to consider ethical issues, such as obtaining informed choice and consent, questions about cost-effectiveness, and the feasibility and practicality of alternative methods to deliver the results. Finally, our approach has focused on monogenic disorders and may require modification to the framework in order to consider other kinds of genomic disorders (such as chromosomal abnormalities) or polygenic risk scores.

## CONCLUSIONS

Actionability includes a broad spectrum of actions that can benefit a person, a family, and society. There is no general agreement on how to define actionability, medical actionability, or clinical utility, and the evidence for monogenic disorders is often of relatively poor quality compared with evidence in other areas of medicine. These factors result in substantial variation in access to and reimbursement for genomic applications. Systematic, standardized, and authoritative assessment of actionability is an important step toward improving population health outcomes through the application of genomic medicine.
